# Acute Poisoning Cases Presented to the Addis Ababa Burn, Emergency, and Trauma Hospital Emergency Department, Addis Ababa, Ethiopia: A Cross-Sectional Study

**DOI:** 10.1155/2021/6028123

**Published:** 2021-12-08

**Authors:** Biruktawit Zemedie, Menbeu Sultan, Ayalew Zewdie

**Affiliations:** Department of Emergency Medicine and Critical Care, St Paul's Hospital Millennium Medical College, Addis Ababa, Ethiopia

## Abstract

**Background:**

Acute poisoning is a common reason for visits to the emergency room and hospitalization across the world, as well as a possible cause of morbidity and death. This study aimed to assess acute poisonings at Addis Ababa Burn, Emergency, and Trauma (AaBET) Hospital. *Methodology*. A one-year cross-sectional study was conducted at AaBET Hospital from February 1, 2018, to January 31, 2019. Data were collected using a structured and pretested questionnaire by the Emergency Medicine and Critical Care residents from acutely poisoned patients' interviews and patient charts.

**Results:**

Data were collected from 98 acute poisoning cases, and 52% were males and 48% were females. 85 (86.7%) were less than 45 years. 52 (55.1%) were unemployed, and 33 (33.7%) were farmers. 96 (98%) cases were due to intentional poisoning, and 96 (98%) had oral ingestion. Organophosphates poisoning (27.5%) was the commonest cause, followed by 26.5% of unknown poisons and 16.3% prescribed drugs. Sixty-six percent of the patients presented to the hospital after 2 hours of ingestion. The case fatalities were 10.2% of which 40% of the cases were due to 2,4-dichlorophenoxyacetic (2, 4-D) poisoning, followed by aluminum phosphide (20%).

**Conclusion:**

This study showed farmers and the unemployed were more affected. The most common mode of poisoning was intentional poisoning, oral ingestion being the primary route. The common poisons used by the victims were organophosphates. 2, 4-D poisoning was the major cause of death.

## 1. Introduction

A poison is any substance that is harmful to the body when ingested, inhaled, injected, or absorbed through the skin. Poisoning can occur intentionally or accidentally as occupational and environmental exposure and also during daily routine activities at home.

According to World Health Organization (WHO) data, in 2012, an estimated 193,460 people died worldwide from unintentional poisoning. Of these, 84% of death occurred in low- and middle-income countries. Poisons such as pesticides and household detergents and drugs such as anticonvulsants, antidepressants, analgesics, antibiotics, and antihypertensives and illegal street drugs of abuse are among the common ones. The WHO estimated that deliberate ingestion of pesticides causes 370,000 deaths each year which makes poisoning a top 50 cause of death worldwide [1].

The pattern of poisoning has been varying according to the geographical location. Compared to developed countries, pesticides and household cleansing agents such as sodium hypochlorite are commonly used poisons in developing countries [2].

A study conducted in three African countries, Botswana, South Africa, and Uganda, stated that acute poisoning has been identified as a significant cause of both morbidity and mortality, and the hospitals' prevalence of poisoning have been known to vary from 1.2% to 17% [3–5].

In Ethiopia, the WHO has estimated that there were 3.5 deaths per 100,000 persons due to unintentional poisoning in 2004 [6]. Poisoning was identified as the second most common method of attempted suicide in a district of Ethiopia. The authors reported that poisoning was used more commonly by women than men, and strong detergents and rodenticides were the most frequently used poisons [7].

This study aimed to assess acute poisoning at the emergency department of Addis Ababa Burn, Emergency, and Trauma Hospital (AaBET).

## 2. Methods and Materials

### 2.1. Study Area and Period

The study was conducted in Addis Ababa Burn, Emergency, and Trauma (AaBET) Hospital, Addis Ababa, Ethiopia, from February 1, 2018, to January 31, 2019. As of November 2021, Ethiopia has a population of more than 115 million. Addis Ababa as the capital city of Ethiopia has a population of more than 5 million. AaBET Hospital is an affiliation of St Paul's Hospital Millennium Medical College (SPHMMC) which is the second top referral hospital in Addis Ababa, Ethiopia. It comprises an Emergency Medicine and Critical Care Department, Orthopedics and Traumatology Department, Neurosurgery Department, Plastic Surgery Unit, and General Surgery Unit since 2014 G.C. The hospital emergency department has 60 beds, 13 Intensive Care Unit (ICU) beds, and 300 inpatient beds. The hospital serves more than 150000 people from Addis Ababa and out of Addis Ababa. The hospital accepts referral patients from 55 health centers and 11 hospitals catchments through liaison communication. The referral is decided by the clinician, availability of further care, and resource in accepting facility. Currently, there is no organized poison center in Ethiopia. Therefore, every health facility provides care for poisoned patients based on their level of service and resources available. The annual emergency room patient visit of the hospital ranges from 15,000 to 20,000. The emergency room and ICU accept all types of cases, while inpatient wards are only for the respective departments and units. Patients requiring medical ward admission will be transferred to SPHMMC.

### 2.2. Study Design

It was a one-year cross-sectional study.

#### 2.2.1. Inclusion Criteria

Patients of all age groups presenting with acute poisoning to the emergency room were included.

#### 2.2.2. Exclusion Criteria

Patients with recreational drugs of abuse (alcohol and cocaine), natural poisons such as stings and envenomation, and food poisoning were not included in the study.

### 2.3. Data Collection Procedure

A structured and pretested questionnaire was used to collect the data from poisoned patients' interviews and patient charts by Emergency Medicine and Critical Care residents after obtaining informed consent. The data collection questionnaire was categorized as sociodemographics (includes age, sex, address, education status, marital status, and occupation); clinical profile (includes triage, referral status, time of arrival, nature of poisoning, mode of ingestion, type of poison, clinical symptoms, previous suicidal attempt, source of poison, and awareness of the complication of the poison); and outcome of patients (length of stay in the Emergency Department(ED) and overall disposition). Sociodemographics and clinical profile (awareness of the complication of the poison) were interviewed from the patient and/or attendant, and the rest were reviewed from patient charts. Data collection did not interfere with patient management. When patients were unable to communicate or give responses, attendants were asked about the circumstances after informed consent and then history was confirmed by the patient after stabilization. Completeness and data quality control were checked by the primary investigator.

### 2.4. Data Analysis

After data were cleared, they were entered into Epi info 3.5.3 and analyzed with SPSS 20. Descriptive statistics were employed and written as numbers, percentages, median, and range.

Ethical clearance was obtained from the SPHMMC ethical review board.

## 3. Results

### 3.1. Sociodemographics

Between February 1, 2018, and January 31, 2019, there were a total of 1543 emergency visits of which 98 (0.6%) poisoning incidents were reported. There were 51 males (52%) and 47 females (48%) among the group. In 59 (60.2%) of the instances, the patients were from Oromia, and 38 (38.8%) cases were from Addis Ababa. 52 people (53.1%) were unemployed, while 33 (33.1%) were farmers (Table 1).

### 3.2. Clinical Profile, Poison Type, and Associated Factors

79 (80.6%) patients were referred from health facilities, while 19 (19.4%) patients came directly to AaBET hospital from home. From those referred, 16/79 (20.2%) of them came within two hours of exposure, and 63/79 (79.8%) of the cases came after two hours of exposure. However, 16/19 (84.2%) patients of the nonreferred cases came within two hours of exposure, and 15 (78.9%) were from Addis Ababa.

32/98 (32.6%) came within two hours of exposure, and 66/98 (67.4%) came after two hours of exposure. The median time of arrival at the hospital was 4 hours, with a range of 1 to 26 hours; 58 (59.2%) triaged to the orange (which requires urgent care) category and 40 (40.8%) to the red side (which requires immediate therapy). Ninety-six (98.0%) were intentional, while 2 (2.0%) were accidental poisonings. 96 (98.0%) were through the oral route, and the remaining two (2.0%) were inhalational.

Overall, eight different groups of poisons were identified. Organophosphates accounted for 27 (27.5%), prescribed drugs 16 (16.3%) which include antibiotics, anticonvulsants (phenytoin, carbamazepine, and phenobarbital), antidepressants (TCA), isoniazid, iron sulfate, and opioids (tramadol), 2, 4-D 9 (9.2%), aluminum phosphide 7 (7%), and others (Table 2).

Fifty-five (56.1%) of the patients had different symptoms of the gastrointestinal system such as nausea, vomiting, dyspepsia, and diarrhea, while 35 (35.7%) had multisystem manifestations. 6/98(6.1%) cases had previous suicidal attempts.

Seventy-three (74.5%) patients got the poison from home, and 25 (25.5%) bought it from drug stores. Seventy-two (73.4%) of the patients and/or the attendants had an awareness of the complications of the poison in humans, including fatalities and long-term sequels.

### 3.3. Outcome of Poisoned Patients

50 (51%) patients stayed for less than 24 hours in the ED, and 48 (48.9%) patients stayed for more than 24 hours in the ED, a maximum of six days. Fourteen (14.2%) patients were admitted to the Intensive Care Unit (ICU). There was no ward admission or transfer to another facility.

76/98 (77.6%) patients were discharged from the ED, while 8/98 (8.2%) patients died in the ED. 88/98 (89%) cases were discharged improved from the hospital, and 10/98 (10.2%) patients died. The details of the patients who died are shown in Table 3.

## 4. Discussion

The majority of the patients who had poisoning in this study were young and adults, especially farmers and the unemployed.

The majority of cases (98.0%) were due to intentional poisoning, similar to other studies, TASH, Ethiopia, 96.5%, and Jimma, Ethiopia, 50.5% [7, 8].

Organophosphate poisoning was most common poison identified. This is similar to previous studies [9–11]. The second most common poison was prescribed drugs, which require proper advice by clinicians and effective follow-up.

The other two commonly identified poisonous chemicals in the study were 2,4-D and aluminum phosphide (AlPO4). It was known that 2,4-D was banned from any use in developed countries.

2,4-D poisoning was responsible for 40% of deaths, while aluminum phosphide poisoning was responsible for 20%.This result showed the need for active preventive strategies in the community. Considering the predominant agrarian community in Ethiopia and free access to chemicals such as pesticides, herbicides, and insecticides, farmers usually stay in the circle and are most affected. Other similar studies also showed the same results [11].

Most of the patients (50%) presented with gastrointestinal symptoms such as nausea, vomiting, diarrhea, and epigastric pain similar to the study in Jimma, Ethiopia (49.5%), which was different from the Tanzanian study where neurologic manifestations (62.3%) were common [9, 12].

Six patients had a previous suicide attempt, and two of the patients had known psychiatric problems. Both of these patients presented with an overdose of their medication.

More than sixty percent of the patients came after the golden hour (the first one to two hours after ingestion), which is different from Gondar, Ethiopia, where ninety percent of patients arrived at the hospital within two hours [8]. This could be because most patients came after referral outside of Addis Ababa and also because of the delay from the referral site. Early arrival at the hospital determines the effectiveness of various decontamination interventions to decrease gastrointestinal absorption, the early initiation of different antidotes, and the provision of the necessary supportive care before decompensation of the vital organs.

According to the study, around 42% of the patients stayed in the emergency department for more than 24 hours, with a maximum of six days and ICU admission for a maximum of 12 days.

10.2% of the patients died of acute poisoning, which is higher than in TASH, Ethiopia (8.6%), and Jimma, Ethiopia (5.8%) [12, 13]. All dead patients presented with severe systemic symptoms involving more than three vital systems, and all came to the hospital after the golden 2 hours of therapy. The three main causes of death were 2,4-D poisoning (40%), almunium phosphide (20%), and organophosphate poisoning (20%). In previous studies in the country, organophosphate was the top cause of death [2, 7, 8, 11, 14].

In this study, all of the patients were approached according to the severity of illness, symptoms at presentation, and toxidrome of presentation. Identification of the poison was carried out by showing some of the samples in the hospital to the patient's families, and some of the families were able to identify the specific poison by name, while others brought the samples to the hospital. Toxicological screening and other necessary laboratory confirmatory tests could not be carried out because of the unavailability of the service. Because of that, around 26% of the poisons were labeled as unknown and 38% of the prescribed drugs could not be identified specifically. These suggest the need for toxicology screening laboratories.

## 5. Limitations

There are several limitations to this study. The first one is that the study was a single-center hospital-based study with a small sample size, which makes it difficult for generalization to the country. The second one is that we depended on clinicians' decisions in cases of poisoning since there were no laboratory tests for toxins. Lastly, further study is required to assess specific management and antidotes for specific poisons in the future since this study mainly assesses the pattern of poisons.

## 6. Conclusions

This study showed age less than 45 years age and males were more affected by intentional poisoning. Organophosphate compounds are the commonest poisoning agents followed by prescribed drugs and 2,4-D poisoning. 2, 4D poisoning followed by aluminum phosphide and organophosphate poisoning were the three main causes of death.

## Figures and Tables

**Table 1:** Demographic data of poisoning patients presented to AaBET hospital.

Variables	Category	Frequency	Percentage

Sex	Male	51	52.0
	Female	47	48.0

Age	13–24	43	43.9
	25–34	35	35.7
	35–44	7	7.1
	>45	13	13.3

Address	Oromia	59	60.2
	Addis Ababa	38	38.8
	Amhara	1	1.0

Level of education	Uneducated	6	6.1
	Primary school	56	57.1
	Secondary school	31	31.6
	College and university	5	5.1

Marital status	Single	56	57.1
	Married	42	42.9

Occupation	Unemployed	52	53.1
	Farmer	33	33.7
	Private business	3	3.0
	Merchant	3	3.0
	Unknown	7	7.2

**Table 2:** Type of poison and associated factors of poisoned patients presented to AaBET hospital.

Variables	Category	Frequency	Percentage

Triage	Orange (urgent)	58	59.2
	Red (critical)	40	40.8

Referral status	Referred	79	80.6
	Nonreferred	19	19.4

Time of arrival	≤2 hours	32	32.7
	>2 hours	66	67.3

Nature of poisoning	Intentional	96	98
	Accidental	2	2

Mode of ingestion	Oral	96	98
	Inhalational	2	2

Type of poison	Organophosphate	27	27.5
	Unknown poison	26	26.5
	Prescribed drugs	16	16.3
	2-4D	9	9.2
	Na Hcl (bleach)	8	8.2
	Al PO4	7	7
	Rodenticides	2	2
	Carbon monoxide	2	2
	Hydrocarbons	1	1

Clinical symptoms	GIS (diarrhea and vomiting)	55	56.1
	CNS (altered mental status)	8	8.2
	CVS and RS (tachycardia, hypotension, and tachypnea)	3	3
	RS and GIS (tachypnea, diarrhea, vomiting, etc.)	19	19.3
	>2 system manifestation CNS, CVS, GIS, and RS^*∗*^	13	13.2

Previous suicidal attempt	Yes	6	6.1
	No	92	93.9

Source of the poison	Home	73	74.5
	Drug store	25	25.5

Awareness on the complication of the poison on humans	Yes	72	73.4
	No	26	26.6

^*∗*^GIS- gastrointestinal system, RS- respiratory system, CVS- cardiovascular system, CNS- central nervous system.

**Table 3:** Death distribution and associated factors of poisoned patients presented to AaBET hospital.

	Gender	Age	Time of arrival to AaBET (hours)	Length of stay (hours)	System affected, GIS, CNS, CVS, and RS^*∗*^	Type of poison

Case 1	M	22	>2 hrs	5 hrs	All 4 main systems	2,4-D
Case 2	M	18	>2 hrs	6 hrs	‘'	2,4-D
Case 3	F	50	>2 hrs	8 hrs	‘'	2,4-D
Case 4	F	14	>2 hrs	3 hrs	‘'	2,4D
Case 5	M	25	>2 hrs	48 hrs	‘'	Aluminum phosphide
Case 6	F	32	>2 hrs	54 hrs	‘'	Aluminum phosphide
Case 7	M	25	>2 hrs	9 hrs	‘'	Organophosphates
Case 8	M	14	>2 hrs	48 hrs	‘'	Organophosphates
Case 9	M	26	>2 hrs	56 hrs	‘'	Unknown
Case 10	M	18	>2 hrs	74 hrs	‘'	Unknown

^*∗*^GIS- gastrointestinal system, RS- respiratory system, CVS- cardiovascular system, CNS- central nervous system.

## Data Availability

The data used to support the findings of this study are available from the corresponding author upon request.

## Abbreviations

AaBET: Addis Ababa Burn, Emergency, and Trauma Hospital
CNS: Central nervous system
CVS: Cardiovascular system
GIS: Gastrointestinal system
RS: Respiratory system
SPHMMC: St Paul's Hospital Millennium Medical College
2,4-D: 2,4-Dichlorophenoxyacetic acid
WHO: World Health Organization.
